# Loading Proteins into Extracellular Vesicles to Camouflage Protein Allergens

**DOI:** 10.1021/acsomega.5c03419

**Published:** 2025-09-08

**Authors:** Estella Rao, Angela Paterna, Valeria Longo, Noemi Aloi, Giorgia Adamo, Sabrina Picciotto, Daniele Romancino, Samuele Raccosta, Antonella Bongiovanni, Paolo Colombo, Mauro Manno

**Affiliations:** † Institute of Biophysics, National Research Council of Italy, via Ugo La Malfa 153, Palermo 90146, Italy; ‡ Institute for Biomedical Research and Innovation, National Research Council of Italy, via Ugo La Malfa 153, Palermo 90146, Italy

## Abstract

Extracellular vesicles (EVs) are cell-derived membranous nanoparticles with a high potential as drug delivery systems due to their intrinsic capability to vehicle biological materials and information. Beyond the transport of small drugs and therapeutics, a biotechnological challenge consists of loading nanoparticles and macromolecules with large size, including antibodies and other proteins. Here, we use microalgal-derived EVs, named nanoalgosomes, that are biocompatible, sustainable, and green EVs derived from microalgae and thoroughly characterized in our previous work, and a recombinant calcium-binding protein (CBP), Par j 4, a minor allergen from *Parietaria judaica* (*Pj*). We set up an efficient method to load Par j 4 into nanoalgosomes, using extrusion as the loading technique, affinity chromatography as the purification method, and fluorescence spectroscopy to quantify loaded cargo. Confocal microscopy is used to show protein and EVs colocalization; specific dot blot demonstrates that loaded Par j 4 is detectable only after EVs lysis, being masked by intact nanoalgosomes. The achieved camouflage of an allergen opens the perspective of addressing an unmet need in the treatment of allergies, that is, the possibility to present allergens in a controlled manner and without side effects. Further, we showed that nanoalgosomes may be efficiently exploited to carry large size macromolecules.

## Graphical abstract

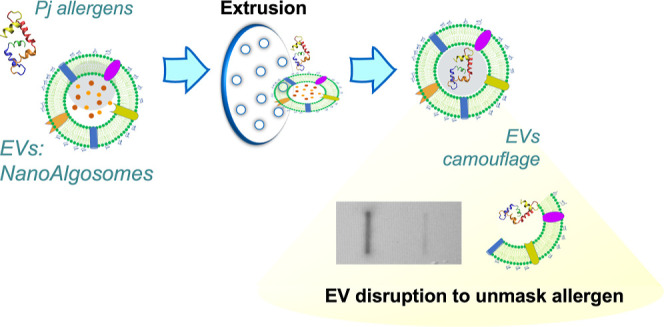

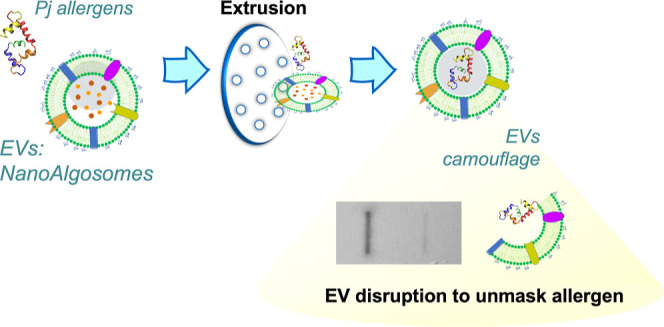

## 1 Introduction

A major therapeutic objective of modern medicine has been the development of novel treatment strategies that can target specific organs, tissues, and cells and deliver bioactive molecules.^1^ As such, a variety of nanoparticle-based drug delivery systems has been tested over the last decades, including synthetic polymer- and lipid-based nanoparticles as well as other nanovectors based on organic and inorganic materials.^2^ Therapeutic agents such as RNA molecules, which are effective in vitro, often fail in vivo due to rapid clearance or biological barriers that prevent site-specific accumulation.^3^ Further, despite the appreciable success of synthetic nanomaterials to date, technical challenges involving their cost-effective production and intrinsic toxicity still hinder their clinical and market translation.^2^

Biogenic nanovesicles, such as extracellular vesicles (EVs), have shown potential to naturally perform cell-specific drug release.^4,5^ EVs are a diverse group of membranous nanoparticles originated from cells involved in several biological processes^6–8^ and recognized as mediators of intercellular signaling and exchange of membrane and cytosolic contents, including proteins and RNA.^9,10^ Moreover, they are naturally stable in various biological fluids, immunologically inert, and able to exhibit organotropic targeting. Specifically, EVs possess a native lipid composition, membrane proteins, and surface glycoconjugates derived from the parent cell, which can facilitate cellular uptake and interaction with biological barriers in a more physiologically compatible manner than synthetic liposomes.^5^ This potential triggered an increasing interest to exploit EVs as therapeutics^11,12^ and in a large variety of biotechnological applications.^13,14^ Cargo can be loaded into EVs by endogenous loading, providing cells with the means to incorporate small molecules/proteins/RNAs into EVs during their biogenesis,^15,16^ or exogenous loading, with incorporation of molecules into or onto isolated EVs by various manipulations.^17,18^

While EVs are secreted by almost all cell types and constitute vehicles for interspecies and cross-kingdom communication,^19^ a growing interest is arising for EVs derived from nonhuman sources, such as bacteria,^20^ bovine milk,^21^ and edible plants,^22–25^ considered as biocompatible, sustainable, green, next-generation nanocarriers.^26^ In such a context, we recently identified microalgae as a novel natural source of EVs, called nanoalgosomes.^27,28^ Beyond the remarkable structural and functional features of microalgal EVs, including their bioavailability, nontoxicity, and nonimmunogenicity,^29,30^ their exploitation as drug carriers is made cost-effective also by the sustainability and scalability of their production.^31^

In this scenario, EVs and in particular nanoalgosomes display all the potentiality to serve as carriers for bioactive large molecules (e.g., allergens) and as a tool for the treatment of immune response dysregulation, such as allergy.^32^ Allergic diseases represent a group of conditions (such as, for instance, chronic respiratory pathologies) caused by hypersensitivity of the immune system to innocuous particles present in the environment, such as dust, mold, or pollen.^33^ One of the most relevant type of pollen allergy in the population living in the Mediterranean basin is *Parietaria judaica* (*Pj*), with a reactivity to its pollen up to 30% of allergic subjects in southern Italy.^34^ The composition of the allergenic extracts of the *Pj* pollen has been extensively studied, and the main protein allergens have been isolated and characterized.^35–37^

Until now, the only causative treatment of allergic diseases, including *Pj*, is the Allergen ImmunoTherapy (AIT), consisting of the repeated administration of allergenic extracts to atopic individuals to modulate their pre-existing immune response. Major issues of the encouraging AIT are due to the standardization of the therapeutic formulations, limited efficacy, potential life-threatening side effects, long duration (3–5 years), and low patient adherence.^38^ A way to bypass these pitfalls could be the use of highly purified recombinant allergens and their coupling to adjuvants to reduce allergenicity and modulate immune response.^39^ Indeed, recombinant allergens are immunologically equivalent to natural allergen extracts^40,41^ and can be produced at low cost in a defined and reproducible manner, increasing the quality and safety of the vaccine. On the other hand, it would be extremely advantageous to embed the proteins into a vector, like a nanoparticle, able to present allergens to cells in a controlled manner without the side effect of a shock reaction. EVs have high biotechnological potential to carry bioactive allergic compounds for specific immunotherapy and mask the allergen from IgE recognition.

Here, we demonstrate that calcium-binding protein Par j 4, a *Pj* allergen, can be loaded into nanoalgosomes and masked to an external detection. In order to induce the protein uptake, we mechanically perturb EV membrane by extrusion, which is a less used loading technique. Also, we adopt a smart and efficient method to purify the final products and remove the unloaded molecules by using affinity chromatography. The overall loading workflow is sketched in Figure 1. The scope of the present work is not to substantiate the application to allergen-specific therapy through in vivo and immunological studies. However, along with the latter nonconventional biotechnological procedures implemented in the current work flow, this work represents a striking proof-of-concept for the capability of EVs, and in particular nanoalgosomes, to operate a camouflage of recombinant allergens. Consequently, it urges further studies to validate the current biotechnology ex vivo and to challenge novel approaches for immunotherapy.

**Figure 1:**
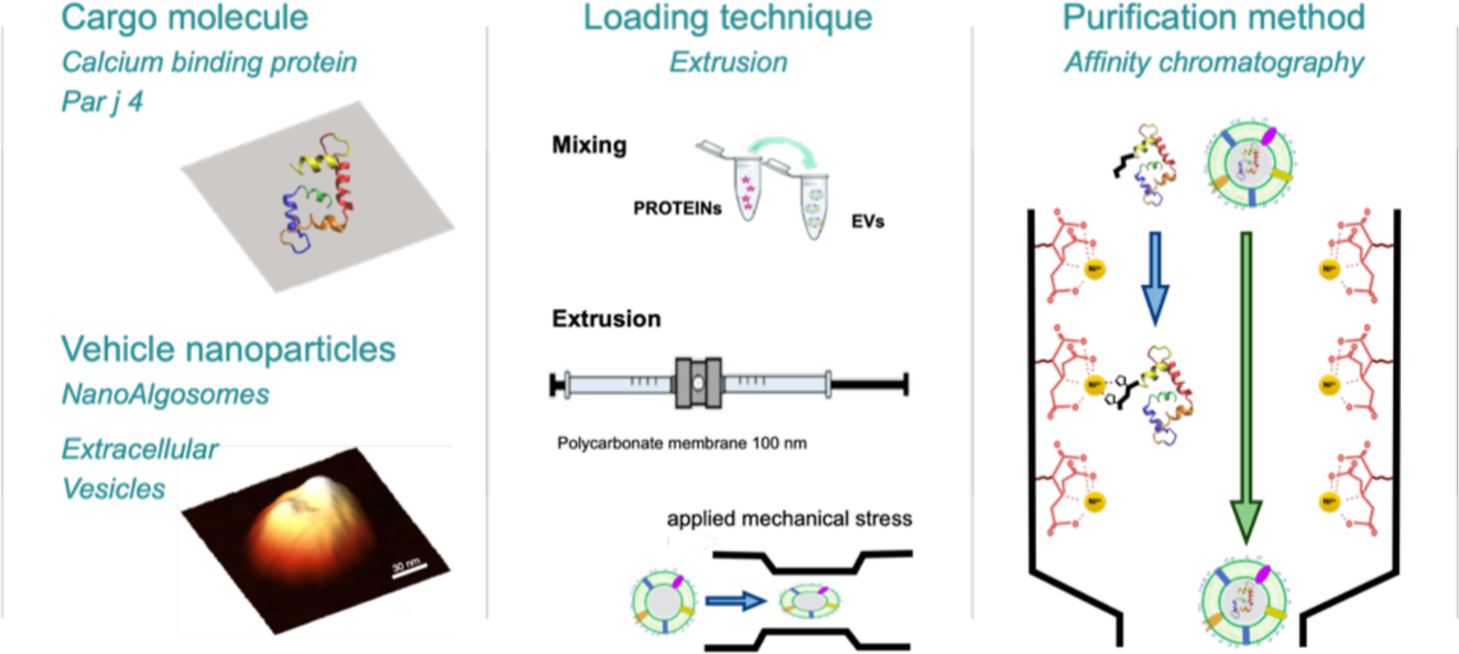
Scheme of the loading bioprocess. Raw materials (proteins and vesicles) are mixed and combined by extrusion. The loaded EVs are purified removing the free protein by affinity chromatography. The nanoalgosome image is a 3D representation of an atomic force microscopy measurement (further details as Supporting Information, Figure S1).

## 2 Materials and Methods

### 2.1 Extracellular Vesicles Production and Isolation

Nanoalgosomes have been isolated from microalgae *Tetraselmis chuii* (*T. chuii*) (CCAP 66/21b) by means of tangential flow filtration, as already described in a study by Paterna et al. 2022.^31^

Specifically, 3 L of microalgal culture was processed, obtaining 5 mL of EV sample diafiltered in PBS. Isolated EVs were aliquoted and stored at 20 °C, in order to perform different experiments using the same batch. Moreover, all results were reproduced by using three independent nanoalgosome batches.

### 2.2 Recombinant Par j 4 Production and Purification

CBP-pQE30 M15 ^36^ colony expressing the full length Par j 4 (96 residues including 12 residues for His-tag and expression, total mass 10.6 kDa) was grown overnight at 37 °C in 100 mL of 2 YT broth (Bacto-tryptone 16 g/L, Bacto-yeast 10 g/L, NaCl 5 g/L, pH 7.0) with 25 μg/mL kanamycin. The cell culture was diluted 1:40 and then was grown in 2 YT with 25 μg/mL kanamycin and 100 μg/mL ampicillin for 3 h at 37 °C. The recombinant Par j 4 expression was induced by adding 1 mM IPTG (isopropyl-d-thiogalactopyranoside) to the culture medium and incubating for an additional 2 h at 37 °C. Cells were harvested and resuspended in a buffer containing 20 mM phosphate buffer pH 7.4 and 0.5 M NaCl, after which they were lysed with mild sonication. Cell debris was removed by centrifugation at 10,000 rpm for 15 min at 4 °C. The supernatant was filtered using a 5 μm disk and then loaded on an HiTrap Chelating HP column (GE Healthcare Biosciences AB, Sweden) following the manufacturer’s instructions. The Par j 4 recombinant protein was eluted using a buffer containing 20 mM phosphate buffer pH 7.4, 0.5 M NaCl, and 500 mM imidazole.

The eluted fractions were analyzed by 12% SDS-PAGE, and the protein purity and concentration were evaluated by Coomassie brilliant blue staining and densitometric analysis (Quantity ONE software, Biorad, USA). Also, the protein concentration was evaluated by absorption spectroscopy taking the extinction coefficients of peptide bond at 205 and 214 nm as 360520 M^–1^ cm^–1^ and 157700 M^–1^ cm^–1^, respectively.^42,43^ Further details are available as Supporting Information (Figure S2).

### 2.3 Protein Labeling

Par j 4 protein was labeled with Alexa Fluor 647 dye (Alexa Fluor 647 NHS Ester, Invitrogen Corporation, Carlsbad, CA, USA). 50 μL of sodium bicarbonate buffer 1 M (pH 8.35) and 200 μL of Par j 4 were added to 100 μg of Alexa647 dye. The reaction solution was stirred for 1 h at room temperature.

High-performance liquid chromatography (HPLC) was performed using a modular Prominence Shimadzu HPLC system (Kyoto, Japan) equipped with an online degasser system (DGU 20A5), a quaternary pump (LC-2010 AT), and an UV–vis photodiode array detector (SPD-M20 A). In order to remove the free dyes from labeled proteins, we performed immobilized metal ion affinity chromatography (IMAC) by using a HisTrap HP 1 mL column (GE Healthcare Life Sciences, Buckinghamshire, UK) with a mobile phase (1 mL/min flow rate) composed of a binding buffer (Dulbecco’s PBS) and an elution buffer (Dulbecco’s PBS + 300 mM Imidazole). A 250 μL sample volume, corresponding to a total protein amount of 250 μg, was injected and equilibrated with the binding buffer; the proteins were then recovered by adding 4.5 volumes (i.e., 4.5 mL) of 300 mM imidazole solution in a one-step gradient. Absorbance was monitored at 650 nm. Subsequently, a buffer change was carried out to remove imidazole. Collected proteins (4.5 mL) were concentrated using 3 kDa Amicon Ultra filters (Millipore) until a final volume of 150 μL and then purified by gravity gel filtration using PD-10 desalting columns packed with Sephadex G-25 resin (GE Healthcare Life Sciences, Buckinghamshire, UK), obtaining 800 μL of Par j 4-Alexa647 at 114 ± 6 μM concentration.

The degree of labeling (DOL) of the final sample was determined by absorption spectroscopy following Alexa647 manufacturer’s data sheet, taking an extinction coefficient at 650 nm for Alexa dye of 239,000 M^–1^ cm^–1^. Since Par j 4 has no aromatic residues, the procedure was modified accordingly, by using the extinction coefficient at 214 nm for Par j 4. Estimated DOL is 17%.

### 2.4 Loading Method

Extrusion was selected as the loading technique. 150 μL of 0.73 nM vesicle solution (0.440 × 10^12^ particles/mL measured by NTA, 49 μg/mL protein content measured by BCA) was mixed with 400 μL of a 52 μM Par j 4-Alexa647 solution and extruded for 31 cycles using a polycarbonate membrane filter with a nominal pore size of 100 nm (Avestin, Manheim, Germany). As control, 150 μL of vesicle solution was extruded with 400 μL of Dulbecco’s PBS and 400 μL of protein solution was mixed with 150 μL of Dulbecco’s PBS. In order to remove the free cargo, a HisTrap column was used to block free protein His-tag. 500 μL of the sample was injected, and the optical density was monitored to assess the EV turbidity (at 214 or 254 nm) and the Alexa absorption (at 630 nm). Flow rate, binding buffer, and elution buffer were used as described in the previous protein labeling section. We collected only the fraction of the sample not bound to the column, that is, the fraction eluting right after sample loading from 0.4 to 2 mL.

### 2.5 Dynamic Light Scattering

Samples (400 μL volume) were pipetted and centrifuged at 1000*g* for 10 min at 4 °C to remove aggregates, if any. The supernatant was thermostated at 20 °C in the cell compartment of a BI200-SM goniometer (Brookhaven Instruments, Holtsville, NY, USA), equipped with a He–Ne laser (JDS Uniphase 1136P, AERI LTD, Bath, UK) tuned at 633 nm and a single-photon avalanche photodiode detector (Hamamatsu C11202–050, Hamatsu, Massy Cedex, France). Measurements were performed as previously described.^31^ The size distribution is computed by assuming for the EVs diffusion coefficient, a Schultz distribution, that is a two parameters asymmetric distribution, determined by the average diffusion coefficient and the polydispersity index PDI.^31^

### 2.6 Nanoparticle Tracking Analysis

EVs size distribution and concentration were determined using a NanoSight NS300 (Malvern Panalytical, United Kingdom). 1 μL of the sample was diluted 1000-fold in SuperQ water, in order to achieve an adequate concentration range for analysis. Measurements were acquired and analyzed as described in a study by Paterna et al. 2022.^31^

### 2.7 Circular Dichroism

Far-UV circular dichroism (CD) spectra were measured by using a J-815 spectro polarimeter (Jasco, Tokyo, Japan) equipped with a Peltier-type temperature-control system. The protein samples were filtered by Amicon Ultra filters (Millipore) 100 kDa in order to remove any aggregates, and 30 μL aliquots were put on a 0.1 mm quartz cuvette for CD measurements. The spectra were acquired with an average of at least 6 scans (3 nm bandwidth, 4 s response, 50 nm min^–1^ scan rate). The buffer solution spectrum was measured as baseline and subtracted from sample spectra. The measured ellipticities θ were converted into the mean residue differential extinction coefficient Δϵ_res_ in M^–1^ cm^–1^ by using the expression $\Delta \epsilon_{res}=\theta {\left(32.982N_{res}dc\right)}^{-1}$ , where *d* is the path length in cm (*d* = 0.01 cm), *N*_res_ = 96. is the number of recombinant Par j 4 residues, and *c* is the protein molar concentration.^44^

### 2.8 Fluorescence Measurements

Fluorescence measurements were carried out at 25 °C in a 1 cm quartz cuvette containing 1.5 mL of sample using a Jasco FP-8500 spectrophotometer equipped with a Jasco ETC-815 Peltier for temperature control. Fluorescence emission spectra of Alexa647 were acquired in the range 605–730 nm with an excitation wavelength of 600 nm. A calibration curve based on the emission at 650 nm was obtained, and the concentration of loaded Par j 4-Alexa647 was quantified, considering the calculated degree of labeling.

### 2.9 Colocalization Experiments

Nanoalgosomes loaded with Par j 4-Alexa647 were stained with Di-8-ANEPPS (ThermoFisher Scientific), a dye that is nonfluorescent in water and strongly fluorescent when incorporated into the lipid bilayer. The staining was performed by incubating at room temperature for 1 h an aliquot of vesicles (5 × 10^10^ particles mL^–1^) and a 500 nM dye solution, previously filtered through 20 nm filters (Whatmann Anotop). Stained loaded nanoalgosomes were imaged using a Leica TSC SP5 confocal laser scanning microscope, with a 63× objective (NA = 1.4). 1024 × 1024 pixel^2^ images were acquired with the sequential acquisition of two channels: DI-8-ANEPPS emission was acquired in the range 518–650 nm with excitation at 488 nm, and Alexa647 emission was acquired in the range 653–750 nm with excitation at 633 nm.

### 2.10 Immunodot Blotting

Immunodot blotting was performed to assess the protein loading into the EVs, using loaded and unloaded nanoalgosomes. Both samples were used without any further treatment as well as after lysis, that is, by adding SDS 0.1% and incubating at 100 °C for 5 min (the process was blocked by putting on ice for 2 min). Additionally, aliquots of the free protein were used as reference control concentrations. 50 μL aliquots of the sample were transferred to a nitrocellulose membrane at different and decreasing final amounts (namely, 10^9^, 10^8^, 10^7^ particles) by using a dot apparatus (slotblot # 80–6095–58 General Electric, USA) and following the manufacturer’s instructions. The membrane was then incubated in 10 mL of blocking solution (Blocker BSA 3% in DPBS-Tween [DPBS w/o Ca^2+^ Mg^2+^, 0.05% Tween 20]) for 1 h at room temperature with shaking. After washing with PBS-Tween, the membrane was incubated for 1 h with HisProbe-HRP (ThermoFisher, Rockford, USA), diluted 1:3000 in DPBS-Tween, and washed four times. The membrane was incubated for 1 min with revealing solution of Pierce ECL (ThermoFisher, Rockford, USA) and then moved in a dark room for film development. After removal of the substrate, membrane was placed in a sheet protector against film and exposed at room temperature for 60 s according to manufacturer’s instructions; then, the film was scanned with an imaging system (Biorad ChemiDoc, Quantity One software version 4.2.1).

### 2.11 Atomic Force Microscopy and Force Spectroscopy

#### 2.11.1 Sample Preparation

Glass slides substrates were derivatized according to the following treatment: (a) they were cleaned by immersion in boiling acetone for a few minutes, dried in a stream of high-purity nitrogen, and activated by UV light (30 W Hg lamp) to expose the hydroxyl groups of silica; (b) they were immersed for 3 min at room temperature in a solution of 0.25 M (3-aminopropyl)-triethoxysilane (APTES) in chloroform, rinsed with chloroform, and dried with nitrogen; (c) they were immersed for 3 min at room temperature in a 0.4 M glutaraldehyde aqueous solution, then rinsed with Milli-Q water, and dried with nitrogen. EV solutions were diluted in PBS to a final concentration of 3 × 10^11^ particles mL^–1^; then, a 30 μL drop was deposited onto APTES/glutaraldehyde functionalized glass slides and incubated overnight at 4 °C in a closed chamber with saturated water vapor pressure to avoid sample evaporation. The samples were gently rinsed with PBS before imaging.

#### 2.11.2 Vesicle Imaging

AFM measurements were carried out in PBS at room temperature by using a Nanowizard III scanning probe microscope (JPK Instruments AG, Germany) equipped with a 15 μm z-range scanner and AC40 (Bruker) silicon cantilevers (spring constant 0.1 N/m, typical tip radius 8 nm). The 2 × 2 μm^2^ images (resolution 256 × 256 pixels^2^) were acquired in the quantitative imaging mode at force set point 160 pN, z-length 50 nm, pixel time 5 ms. The cantilever was thermally calibrated by using the tool in JPK software.

#### 2.11.3 Force Spectroscopy

Force curves were collected by the Force Mapping tool using the same AC40 tip, a maximum force of 320 pN, and a Z speed of 2 μm/s. JPK data processing software (version 6.0.63, Bruker Nano GmbH, Berlin, Germany) was used to perform the elasticity fit in the extended parts of the force–distance plot, after baseline subtraction, contact point determination, and tip–sample separation calculation. The Young’s modulus (*E*) was calculated for each curve of force versus indentation by using the Hertz-Sneddon model and considering a half angle to edge α = 10° of a triangular pyramid indenter and a Poisson’s ratio ν = 0.5.

## 3 Results and Discussion

### 3.1 Raw Biomaterials. (I) Microalgae-Derived EVs (Nanoalgosomes)

EVs were isolated from the marine chlorophyte microalgae *Tetraselmis Chuii* (*T. Chuii*) that we have identified as one of the most promising bio resources for a large-scale production of EVs.^27,28,31^ Upstream conditions were tuned to grow the microalgal strain in medium-scale (3–10 L) photobioreactors under axenic conditions using a batch scalable cultivation method.^31^ The harvested culture was processed by the downstream procedure we have previously optimized and based on tangential flow filtration (TFF).^31^ In brief, sequential TFF steps were performed by using fibers with different cutoffs: (i) clarification from cellular biomass (650 nm cutoff), (ii) isolation of EVs (200 nm cutoff), and (iii) diafiltration and volume reduction to remove small molecules, transfer in the final buffer, and concentrate (20 nm cutoff).

In the end, the overall bioprocess was validated by using a selection of minimal information studies on EVs (as in MISEV guidelines^45^), including colorimetric BCA assay for the protein content, nanoparticle tracking analysis (NTA) for the number concentration, dynamic light scattering (DLS) for the size distribution, atomic force microscopy (AFM) for the morphological features, and immunoblotting of specific EV markers to assess EV identity^27,30^ (further details as Supporting Information, Figure S1).

For microscopy observations, EVs were stained by using Di-8-ANEPPS, a fluorescent dye exhibiting a visible emission spectrum when associated with the lipid bilayer.^27^

Even if in compliance with MISEV guidelines, it is worth considering that an isolation process starting from a cellular culture may include cosolutes of different types and end-up in an EV-enriched secretome.^8^ Such possible occurrence on one side may impair the development of a mature manufacturing process but on the other side, it is not detrimental to the purpose and to the function of the engineered products.^8^

### 3.2 Raw Biomaterials. (II) Calcium-Binding Protein Par j 4

The protein Par j 4, which is a calcium-binding protein (CBP), is a minor allergen of *P. judaica*. The two major allergens are Par j 1 and Par j 2, belonging to the nonspecific lipid transfer protein (nsLTP) family. All these allergens have been previously isolated and biochemically and immunologically characterized.^35,36,46,47^ Here, we focus on the Par j 4 allergen that we know to be stable in solution and we can produce with high yield. We express it in *E. coli* as recombinant protein containing an amino-terminal hexa-histidine tag and purified by immobilized affinity metal chromatography (IMAC). A small fraction of protein aggregate is detectable by DLS after the first purification step, and it was easily removed by 100 kDa centrifuge filters (further details are given in Supporting Information, Figure S2). Eventually, protein was labeled with Alexa647, an available commercial dye able to bind with primary amine groups, with a high brightness (quantum yield) and photostability in different cellular environments.

### 3.3 EV Loading. (I) Loading Technique: Extrusion

Different active methods are currently available to perform the exogenous loading of small molecules and RNA.^17,48^ These include: (i) freeze–thaw, a method relying on a mild loosening of bilayers interaction through temperature cycles,^49^ which is not expected to be efficient for nanoalgosomes that are quite stable even well above room temperature;^27^ (ii) sonication and electroporation, by which molecules can be induced to cross the EV membrane by forming large pores under either acoustic (sonication) or electric (electroporation) perturbation and with the possibility of a sample damage or precipitation due to the high energy transmission;^50–52^ (iii) saponin treatment, a promising method to enhance membrane permeability by using saponins;^53–55^ (iv) lipofectamine transfection, another promising method in terms of high loading efficiency,^56,57^ which on the other hand implies the fusion with exogenous lipid components that may alter the properties of membrane bilayer; (v) extrusion, a method consisting in applying a mechanical perturbation by several passages through a filter membrane, that has been extensively used to coat nanoparticle with EVs membrane more than to encapsulate large macromolecules.^49,58,59^

Here, we used the latter method since the noticeable mechanical perturbation implied by such a procedure should also guarantee high loading efficiency in the case of proteins and more generally of large size macromolecules.

We leverage on the structural robustness of nanoalgosomes, which are stable even above room temperature and might not respond efficiently to freeze–thaw cycles.^27^ In addition, sonication and electroporation, while potentially favoring internalization, can result in significant sample damage, protein denaturation, or EV aggregation. Although extrusion does not create pores in the same way, it does impose strong mechanical stress (via shear and compression) able to transiently loosen membrane structures without fully disrupting them.

Both the proteins and the EVs are mixed and then extruded by 31 passages through a membrane with a 100 nm pore size, which is larger than or comparable to the size of EVs. The methods is operatively similar to the one typically used to build liposomes.^60^ However, for already formed EVs, it is plausible that the net effect is not to disassemble the lipid bilayer but to apply compression or shear forces that loosen the membrane allowing the encapsulation of the exogenous cargo (Figure 1).

### 3.4 EV Loading. (II) Purification Method: Affinity Chromatography

In any EV loading procedure, an important and often overlooked step is the removal of free molecules after complex formation. Since the cargo molecule is typically mixed in large excess (several orders of magnitude) with respect to the large nanoparticles, methods based on sedimentation or osmotic equilibria, such as (ultra)­centrifugation or dialysis, are hardly efficient. For the same reason, sequential filtration or even diafiltration may be not only time-consuming but also insufficient. Size exclusion chromatography may be an adequate solution since the sizes of the molecular cargo and the EVs are quite different. Here, we implemented another very efficient method using immobilized metal ion chromatography (IMAC) and leveraging on the presence of a His-Tag in the recombinant proteins.^52^ First we verified that nanoalgosomes have no affinity for Nichel ions and can pass through HisTrap columns with complete recovery. In Figure 2, the same EV sample is injected into the HPLC system using both no column and the HisTrap column. The presence of a 1 mL column of course delays the sample elution but does not affect the complete release of the sample; indeed, the area of the peak obtained with no column is equal to the area obtained after the elution through the column, indicating that no sample loss occurs (the peak areas were calculated by using General Public License software, XMGRACE, https://plasma-gate.weizmann.ac.il/Grace). Moreover, control measurements were performed to confirm that extruded Par j 4 preserves affinity with the column and is retained by the HisTrap column (further details are given in Supporting Information, Figure S3). Therefore, we used IMAC to purify loaded EVs from the free protein: the extruded protein-EV samples were injected into the HisTrap column; then, the loaded nanoalgosomes are collected from 0.4 to 2 mL (as for unloaded nanoalgosomes in Figure 2), while the free His-tagged proteins are immobilized in the column, as in the scheme of Figure 1. The collected sample was then concentrated by ultracentrifugation (at 118,000 g) up to a final concentration of 0.23 × 10^12^ EVs mL^–1^ (0.38 nM).

**Figure 2:**
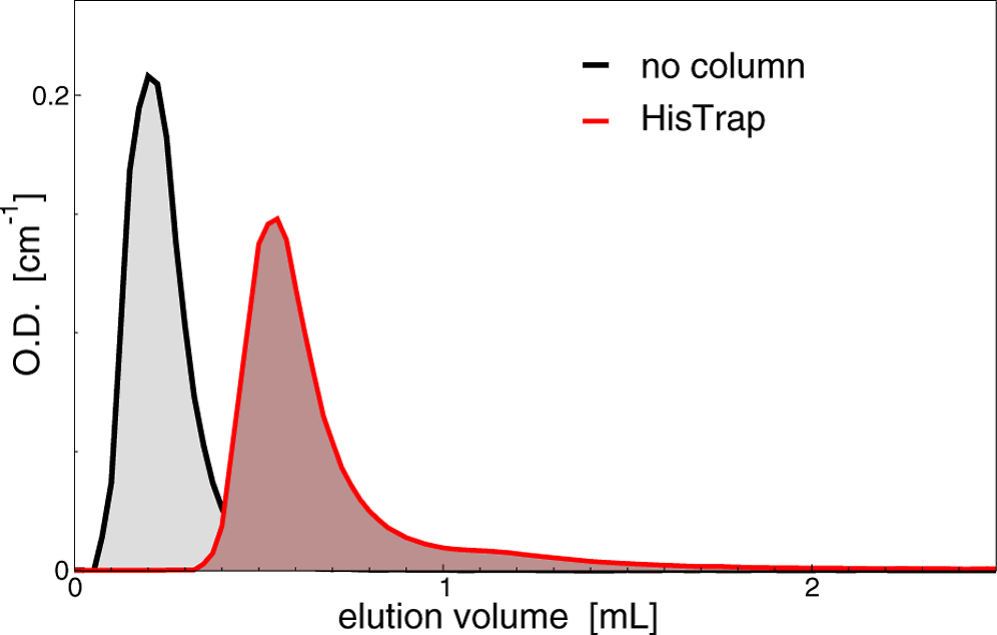
Chromatograms of the same nanoalgosome sample eluted without or with the HisTrap column. Absorbance at 254 nm is shown.

### 3.5 EV Loading. (III) Cargo Quantification: Fluorescence Spectroscopy

Protein labeling allows for the quantification of loaded Par j 4. It is a useful procedure when the total loaded cargo is too low to be revealed by other techniques, such as UV–vis spectroscopy. Exploiting the fluorescence emission intensity of Alexa647 dye, it is possible to obtain a calibration curve and determine the amount of protein loaded into extracellular vesicles, starting from the emission intensity of the loaded nanoalgosomes and the degree of labeling resulting from labeling process. As shown in Figure 3, the emission spectrum of loaded EVs suggests that the amount of encapsulated Par j 4 corresponds to 82 nM in 0.23 × 10^12^ EVs mL^–1^, that is 215 proteins per vesicle. As a comparison, we may consider that more than 5000 proteins of about 3.3 nm may be contained into the lumen of an ideally spherical EV of about 75 nm in size, and more than 1500 proteins are needed to complete the EV’s corona.

**Figure 3:**
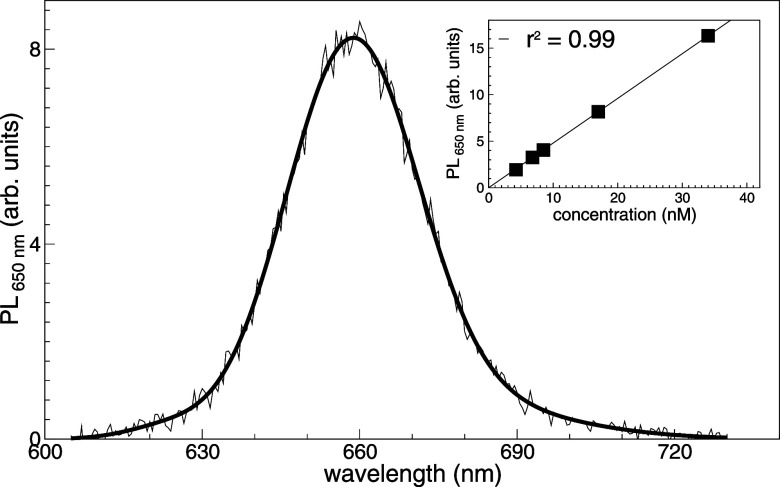
Fluorescence spectrum of loaded EVs. Spectrum has been acquired at 25 °C in the range 605–730 nm, with λ_exc_ = 600 nm. The calibration curve of Alexa647 is shown in the inset, plotting the intensity of the fluorescence at 650 nm versus the concentration (nM) of Alexa647.

It is important to note that the emission spectrum and fluorescence quantum yield of the dye may change depending on the environment. For instance, in a lipid bilayer, the fluorescence intensity of Alexa647 maybe damped from 10 to 40 times^61^ with respect to intensity in a aqueous environment. In the present experiments, we cannot univocally determine the actual environment of the Alexa647 dye: the loading process may bring the stained proteins to interact with the lipid bilayer, with the EV lumen, or even with the external corona; moreover, the dye is bound to the protein itself in different amino acidic residues. In summary, the loading efficiency estimated by fluorescence should be considered as a lower limit, and one may take into account a possible, if not probable, underestimation by even an order of magnitude.

### 3.6 Loading Controls. (I) Structural Integrity

The biophysical properties of loaded nanoalgosomes were compared to those of raw unloaded ones. The size distribution was largely unaltered by loading, as observed by DLS distributions (Figure 4a) and by NTA distributions (Figure 4b); noteworthy, the latter ones are biased toward larger objects as expected due to the intrinsic characteristics of the technique; nevertheless, the two distributions can be completely superimposed.

**Figure 4:**
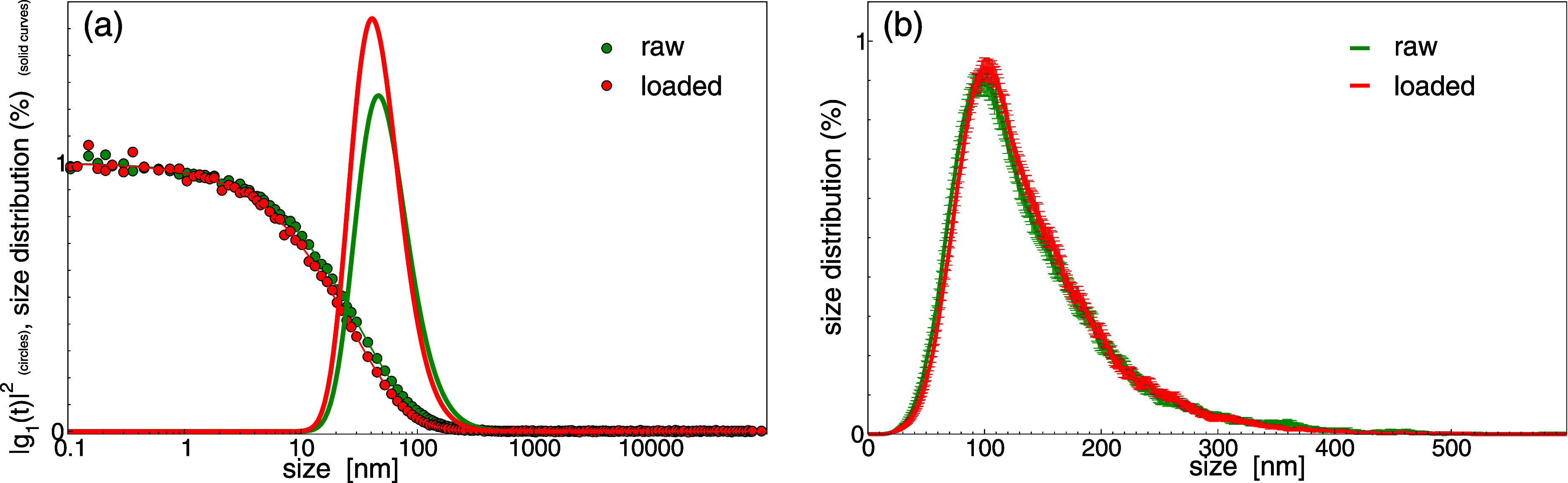
(a) DLS autocorrelation functions and related size distributions for raw (green circles and lines) and loaded EVs (red circles and lines); (b) NTA size distributions for raw (green lines) and loaded EVs (red lines). The area of both distributions is normalized to 100.

The EV morphology observed by atomic force microscopy (AFM) was not altered by the loading procedure (Figure 5a,b). Also, the biomechanical properties were unchanged, as assessed by atomic force spectroscopy (AFS), that is, the AFM used to acquire the intensity of the force probed while approaching the deposited object. By AFS, we measure the Young’s modulus, that is a main biomechanical property of biological matter, related to the compressive stiffness (Figure 5c,d).^62^

**Figure 5:**
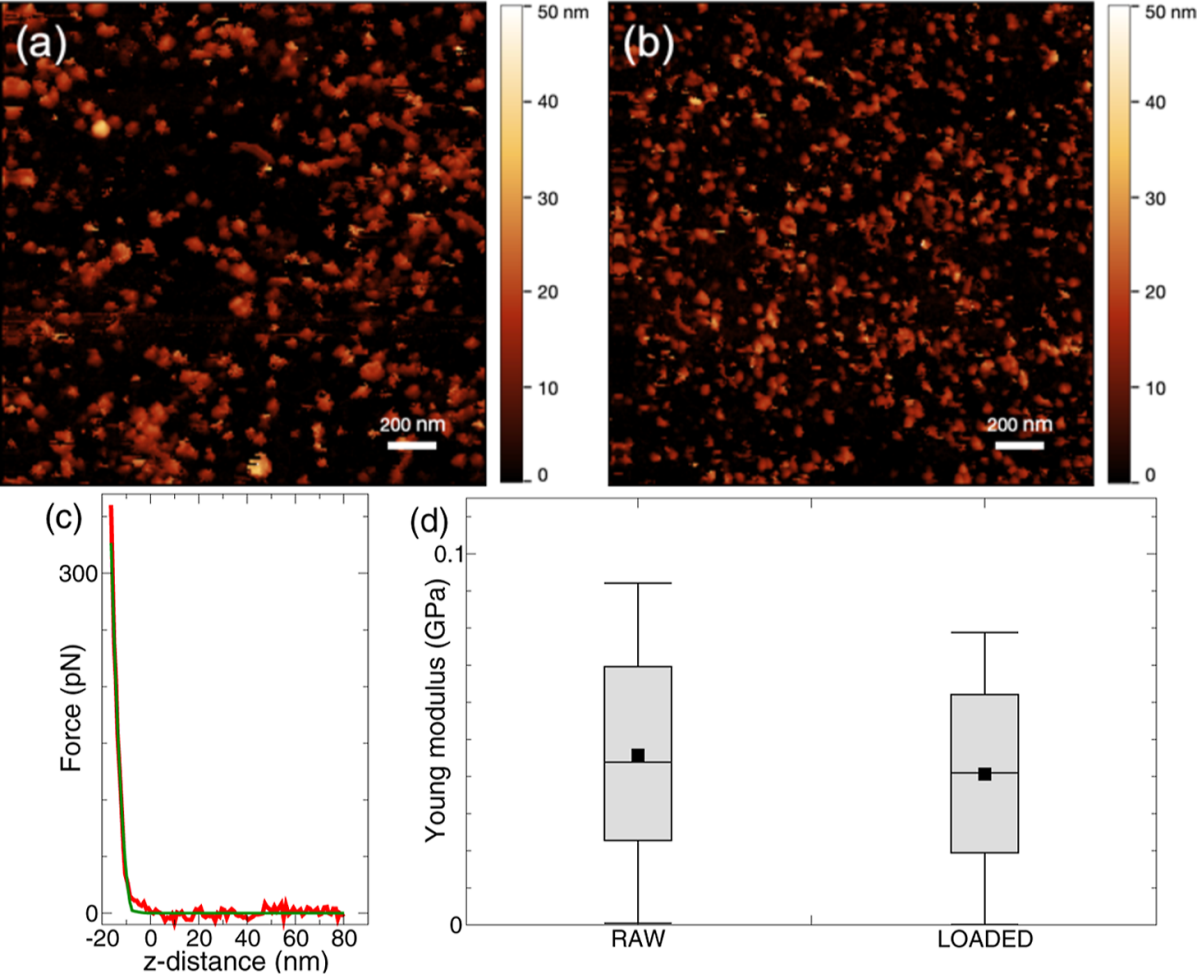
Biophysical control on raw/unloaded and Par j 4 loaded nanoalgosomes. (a) AFM image of raw EVs. (b) AFM image of loaded EVs. (c) Typical force response of AFM when approaching a vesicles (red line) and the best fit by assuming a Hertz-Sneddon model (green line). (d) Young modulus of unloaded and loaded EVs: the box whiskers and edges mark the 100%, 75%, 50%, 25%, and 0% percentile; the black dot indicates the average.

In addition, if the mechanical perturbation may change EVs structural organization, one cannot exclude that it may also affect the stability of loaded proteins. Indeed, we preliminary checked that the protein structure was not altered by extrusion, as shown by the CD spectra of raw and extruded Par j 4, which largely maintains the native conformation (Figure 6). The typical α-helical folding conformation of Par j 4 is assessed by circular dichroism (CD) spectra at 20 °C, while at high temperature, the conformation is slightly relaxed (Figure 6).

**Figure 6:**
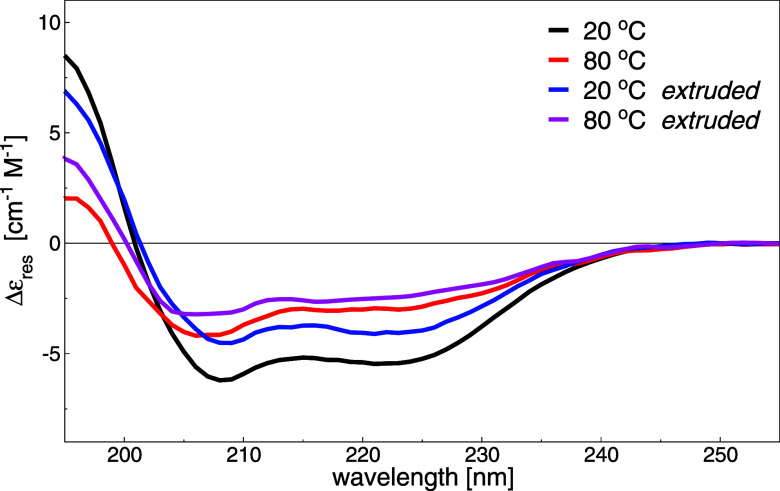
CD spectra of Par j 4 at different temperatures and after mechanical extrusion.

IgE binding to allergens plays a pivotal role in the allergic response, and this interaction is heavily influenced by the 3D structure of the allergen. Notably, most allergenic epitopes are conformational, emphasizing the importance of preserving the allergen structure during experimental and therapeutic processes. Our results demonstrate that loading does not alter the structure of the allergen, preserving its native conformation. This finding is crucial to ensure the integrity of the allergen in future medical applications.

### 3.7 Loading Controls. (II) Colocalization

In order to investigate the colocalization of labeled Par j 4 and nanoalgosomes, Di-8-ANEPPS dye was used to stain EVs. A colocalization of protein and vesicles results in the overlap of the fluorescence emission coming from the two dyes. Representative confocal microscopy images of loaded EVs are shown in Figure 7. The green signal is derived from Di-8-ANEPPS incorporated into nanoalgosomes, while the red signal corresponds to Alexa647, the dye used to label Par j 4 proteins. Control measurements were acquired to confirm that the green fluorescence signal is due to the insertion of the dye in the vesicle lipid bilayer and not to an interaction between the dye and the protein (further details are given in Supporting Information, Figure S3). Panels (a–c and d–f) are related to different sample areas with different magnification. The higher magnification (panels d–f) enhances the colocalization of the two signals. Observed spots differ from the sharp membrane contours typically observed in larger vesicles or in higher-resolution settings. This appearance likely results from a combination of the subdiffraction size of the vesicles, the uniform membrane staining by Di-8-ANEPPS, and the limitations of confocal resolution. The signal is not concentrated at a distinct contour but appears more diffuse due to the optical blurring of small spherical vesicles close to or below the diffraction limit. The microscopy images highlight the absence of large protein aggregates and, at the same time, show a few colocalization spots that cannot be used to quantitatively estimate the loading efficiency. Indeed, within each vesicle, the fluorescent signal is originated by a limited number of fluorescent proteins, so that one may expect to detect only the EVs loaded with the highest efficiency. The heterogeneity of loading, that is, the not uniform distribution of cargo in the EVs, is actually pointed out by the observation of a few spots. Beyond these limitations, confocal microscopy images were important to qualitatively confirm the presence of loaded EVs. Future studies will require the use of super-resolution microscopy in order to assess in greater depth the position of the proteins with respect to vesicles.

**Figure 7:**
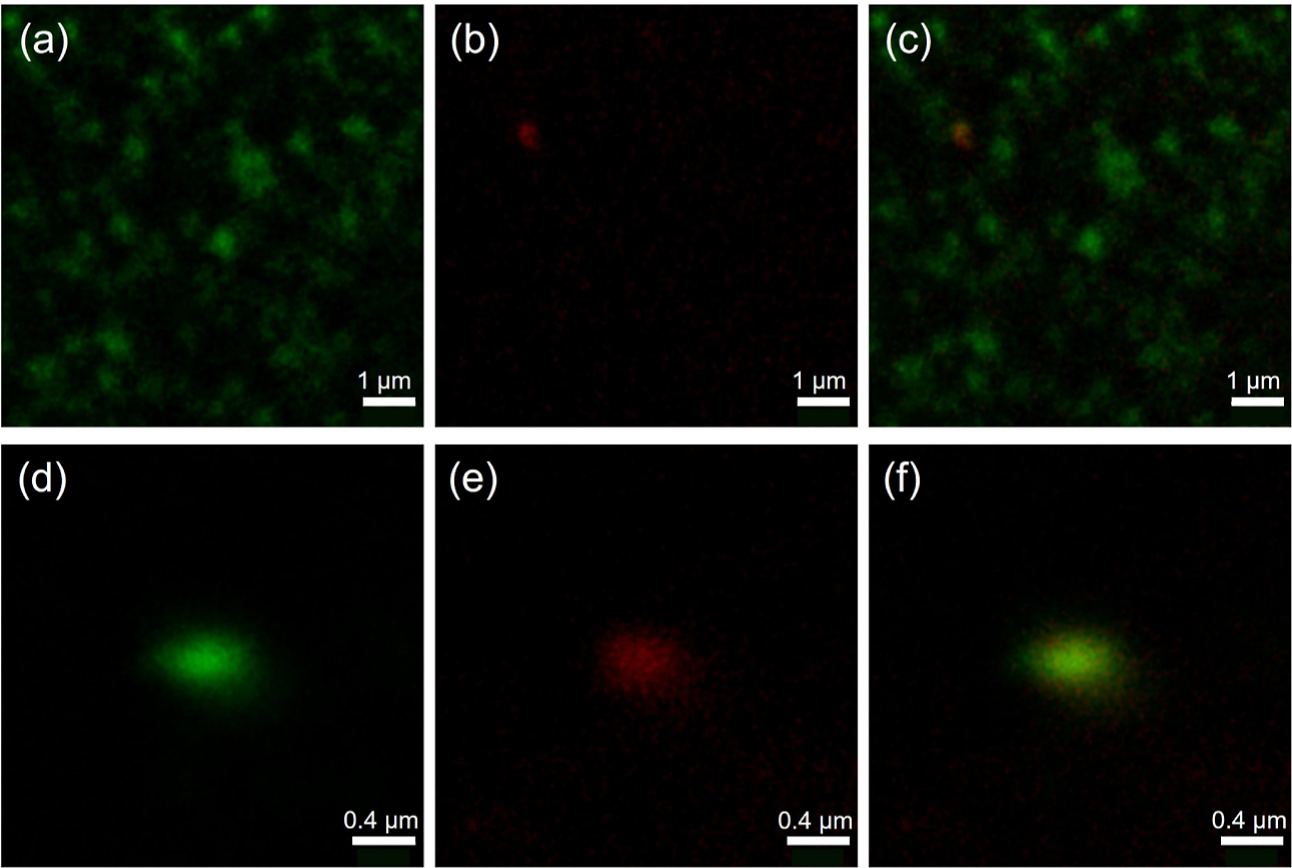
Representative confocal images of Par j 4 loaded nanoalgosomes. (a,d) Di-8-ANEPPS fluorescence emission channel, acquired in the range 518–650 nm with λ_exc_ = 488 nm (green signal); (b,e) Alexa647 fluorescence emission channel, acquired in the range 653–750 nm with λ_exc_ = 633 nm (red signal); (c,f) merge of the two channels. (a–c) Scale bar 1 μm; (d–f) Scale bar 0.4 μm.

### 3.8 Loading Controls. (III) Camouflage

The nanoalgosomes loaded with Par j 4 can mask the presence of the protein. This was elicited by the fact that Par j 4-EVs are able to escape IMAC controls, that is, to pass through the His Trap column without any affinity, while both the protein or protein aggregates are retained in the column.

In order to definitively confirm the loading of Par j 4 into nanoalgosomes, we performed dot-blot experiments on integer and lysed EVs (Figure 8). More specifically, we used a chemiluminescent probe specific for the His-tag. The probe is able to detect the proteins at different concentrations, while it is not able to detect them when applied to intact loaded vesicles. When we disrupt the EVs upon boiling in the presence of detergents (10 min at 100 °C with 0.1% SDS), Par j 4 is released and observable. This is striking evidence that the loading procedure is able to mask the protein, or at least the N-terminus His-tag, from external probes.

**Figure 8:**
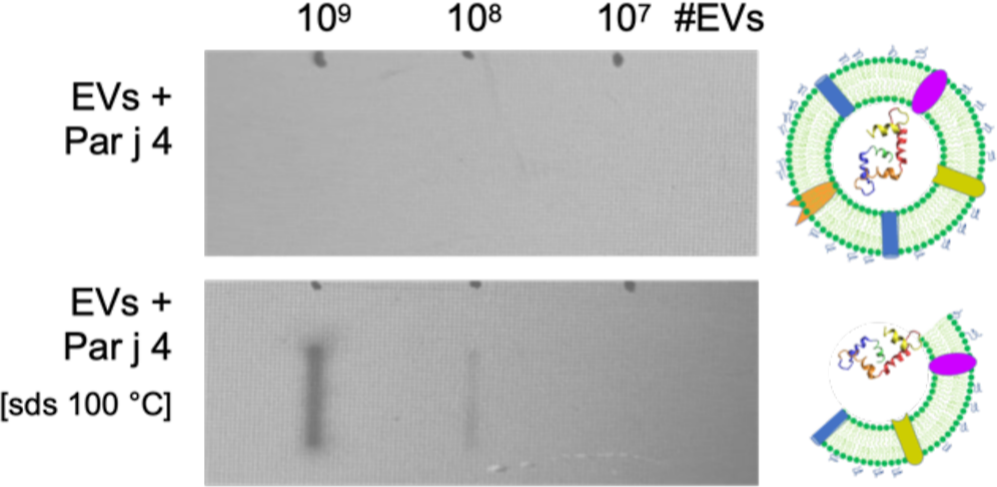
Immunodot blotting of Par j 4-loaded nanoalgosomes at different concentrations. Top stripe: Par j 4-loaded EVs without further manipulation; bottom stripe: Par j 4-loaded EVs after disruption by incubation at 100 °C with 0.1% SDS. A representative cartoon is shown on the right of each stripe.

Control measurements were performed in order to assess that the amount of revealed protein is independent of the specific treatment (further details are given in Supporting Information, Figure S3).

## 4 Conclusions

Safe, efficient, and specific nanodelivery systems are essential to therapeutic intervention and current precision medicine. The effort of translation into clinical application of EV-based therapy may also promote the development of novel technologies and will allow clinical scale EV isolation-purification to be transferred to GMP production according to European legislation.^11,12,63^ Here, we showed a clear proof of concept for the possibility of camouflage a protein by using EVs. In particular, (i) we demonstrated the capability of nanoalgosomes, a promising EV subtype obtained from a sustainable green source, to be loaded with large-size molecules and in particular with proteins; this is still a challenging goal, if one considers that exogenous (postharvesting) loading of proteins into EVs has been rarely achieved,^49,56,64^ while protein loading is currently performed by engineering the progenitor cells^15,16^ and by exploiting protein–membrane interactions to enhance molecular sorting into EVs;^65,66^ (ii) we implemented the extrusion technique as a method to apply high mechanical perturbations without disrupting vesicles integrity; while extrusion is a well-established method for the assembly/disruption of synthetic or cellular membrane, it is still marginally used for loading of extracellular vesicles and it cannot be considered a standard procedure;^49,58,59^ (iii) we applied a smart method for unloaded cargo removal by exploiting the affinity properties of the molecule itself (the method was already used with EVs but not for protein cargo^52^); (iv) eventually we highlighted how a protein allergen can be masked with respect to the external probe. As a matter of fact, we cannot assess where the protein is located in the EV and if it is actually encapsulated within the vesicle lumen or rather entrapped close to the vesicle membrane. Indeed, further studies are required to test all of these hypotheses. However, it is worth noting that a major priority in allergen immunotherapy (AIT) is safety as patients are allergic to the very active principle used in the vaccine and may experience systemic reactions due to pre-existing allergen-specific IgE antibodies on effector cells. Therefore, the possibility itself to operate a camouflage of allergens, reducing the uptake and presentation of free allergen^67,68^ and dampening the immune response, opens new perspectives to enhance the safety profile of therapeutic formulations in immunotherapy and to envisage further clinical or industrial translations. Also, nanoalgosome are nontoxic and nonimmunogenic particles^30^ and are therefore a suitable candidate for therapeutics application. The validation of these results in ex vivo and clinical studies will be a small further step for future studies and hopefully a giant leap for allergen immunotherapy.

## Supplementary Material



## Notes

Additional experimental details concerning characterization of raw EVs, characterization and labeling of Par j 4, and loading controls (PDF)
