# Supplementary material for: Loading Proteins into Extracellular Vesicles to Camouflage Protein Allergens
Source: ACS Omega. 2025 Sep 8;10(36):41029–39. doi: 10.1021/acsomega.5c03419 (PMC12444598; doi:10.1021/acsomega.5c03419)
Supplement: Supplementary file 1 [file ao5c03419_si_001.pdf]

— Supporting Information —

Loading proteins into extracellular vesicles to camouflage protein allergens

Estella Rao,[†,¶] Angela Paterna,[†,¶] Valeria Longo,[‡] Noemi Aloi,[‡] Giorgia Adamo,[‡] Sabrina Picciotto,[‡,†] Daniele Romancino,[‡] Samuele Raccosta,[†] Antonella Bongiovanni,[*,‡] Paolo Colombo,[*,‡] and Mauro Manno[*,†]

†*National Research Council of Italy, Institute of Biophysics, via Ugo La Malfa 153, Palermo, 90146, Italy;*

‡*National Research Council of Italy, Institute for Biomedical Research and Innovation, via Ugo La Malfa 153, Palermo, 90146, Italy;*

¶*These authors contributed equally to this work*

E-mail: antonella.bongiovanni@cnr.it; paolo.colombo@cnr.it; mauro.manno@cnr.it

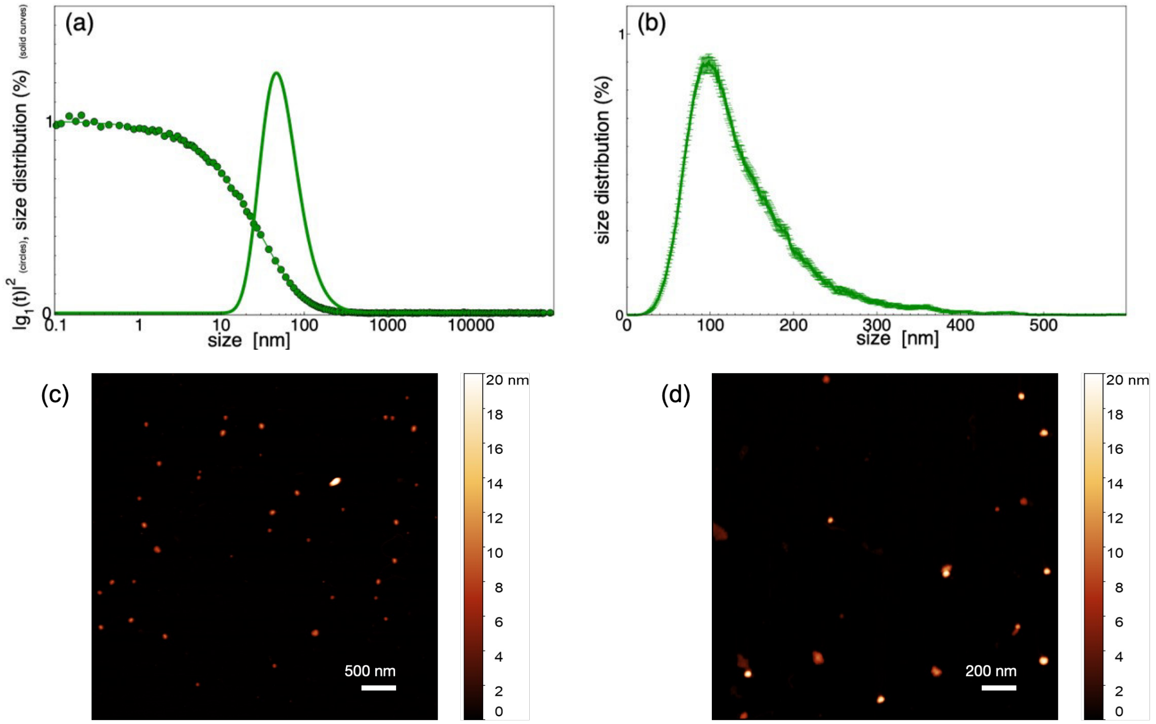

(e) EVs characterisation

Protein content [μg/ml]	49 $\pm$ 4
Concentration [10^9 particles/mL]	440 $\pm$ 20
Z-average size [nm]	65 $\pm$ 5
PolyDispersity Index	0.33 $\pm$ 0.01

(f)

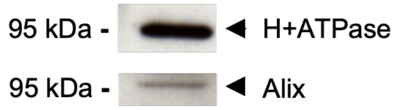

Figure S1: Characterisation of raw EVs.
(a) DLS autocorrelation functions (green circles) and related size distributions (green solid line) for raw EVs.
(b) NTA size distributions for raw EVs (the area of both distributions is normalised to 100).
(c)-(d) AFM images of raw EVs at different scale (as in the white bar). A 40 ul drop of raw vesicles at concentration 2 x 10^{10} particles mL^{-1} were deposited on freshly cleaved mica for 20 minutes at room temperature and dried by nitrogen flow and tapping mode AFM images (512x512 pixel for panel c and 1024x1024 pixel for panel d) were acquired in air by using a NSC-15 (Mikromasch) cantilever (spring constant 40 N/m, typical tip radius 8 nm) as described in Paterna et al. 2022, Front. Bioeng. Biotechnol. 10:836747, doi: 10.3389/fbioe.2022.836747.
(e) EVs characterisation: Protein content determined by BCA; Particle number measured by NTA; Z-average size and PolyDispersity Index measured by DLS. All characterisation measurements have been performed on three different EVs batches.
(f) Immunoblot analyses were performed on raw nanoalgosomes to detect the markers H+ATPase and Alix, as described in Paterna et al. 2022, Front. Bioeng. Biotechnol. 10:836747, doi: 10.3389/fbioe.2022.836747. Representative results of three independent biological replicates are presented.

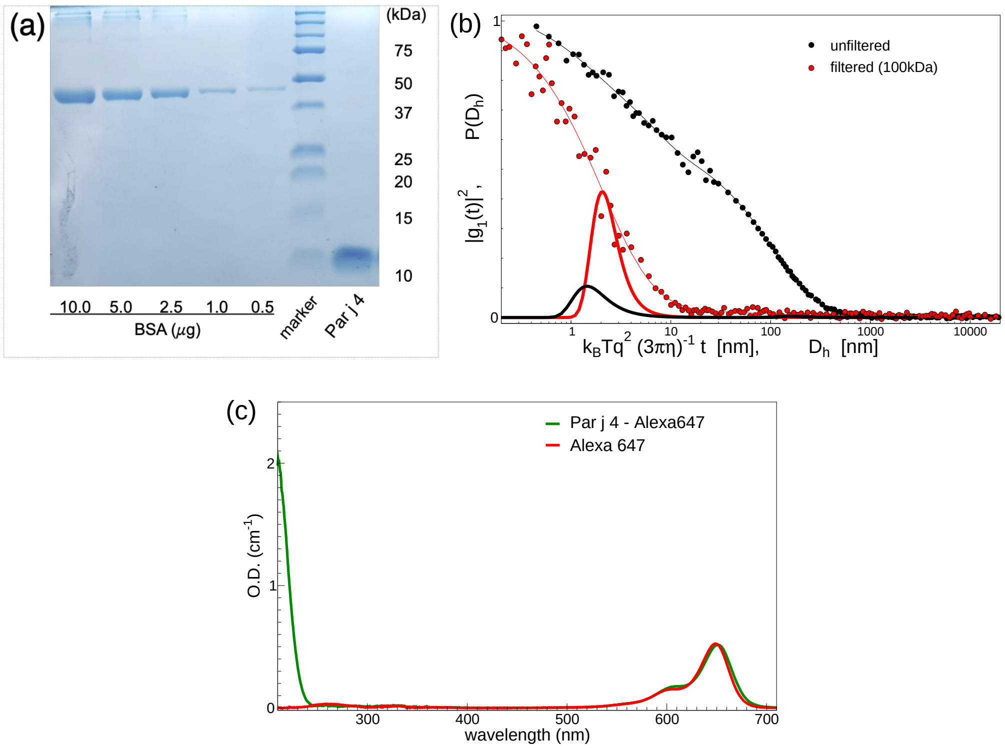

Figure S2: Characterisation and labeling of Par j 4.
(a) SDS PAGE (polyacrylamide gel electrophoresis). Lanes 1-5: BSA at different concentrations as in the panel axis; lane 6: protein markers of different molecular weight as in the right axis; lane 7: Parj4.
(b) DLS autocorrelation functions $|g_1(t)|^2$ and related distribution $P(D_h)$ of size, namely hydrodynamic diameter D_h. The aggregate fraction is removed after 100 kDa filtration.
(c) Absorption spectra of labelled Par j 4 and free Alexa647.

(a) EVs characterisation

	raw EVs	loaded EVs
Protein content [μg/ml]	49 $\pm$ 4	21 $\pm$ 2
Concentration [10^9 particles/mL]	440 $\pm$ 20	235 $\pm$ 16
Z-average size [nm]	65 $\pm$ 5	55 $\pm$ 5
PolyDispersity Index	0.33 $\pm$ 0.01	0.30 $\pm$ 0.01

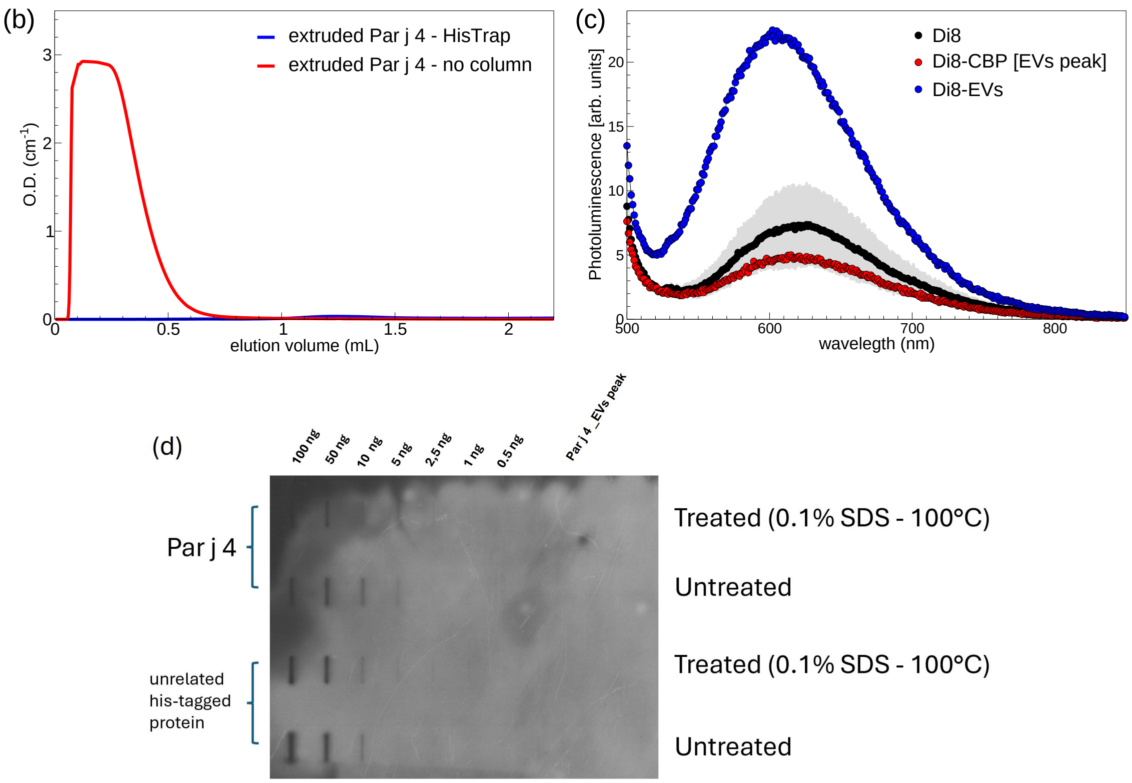

Figure S3: Loading controls.
(a) raw and Par j 4 loaded EVs characterisation parameters. (b) Chromatograms of extruded Par j 4 (Absorbance at 214 nm). The same sample of extruded proteins is injected into HPLC system using both no column (red line) and the HisTrap column (blue line). As in figure 2, when no column is included the sample is immediately eluted; at the opposite when passing through HisTrap columns it is retained in the column since no signal is detectable in the fraction from 0.4 to 2 mL, when loaded EVs are collected. (c) Fluorescence spectra of DI-8-ANEPPS dye in different conditions. The dye was added to samples of extruded Par j 4 (red dots) and extruded EVs (blue dots) eluted with affinity column and collected between 0.4 and 2 ml, at the same concentration and EVs/dye ratio used for confocal microscopy experiments (section 2.9). The spectra of DI-8-ANEPPS diluted in PBS were taken as a blank (black dots and grey shadow); here the shadow marks the actual variability of the dye signal when dissolved in aqueous solutions. This figure compares the signal due to DI-8-ANNEPS staining extruded (or loaded) EVs (blue dots) with the signal due to DI-8-ANNEPS added to the sample eluted in the same time slot of EVs after injection of a sample of extruded proteins (red dots). The equivalence of the latter signal with the dye signal in PBS exclude any interference due to residual protein objects, if any, in the loaded EVs sample. (d) Immunodot blotting of his-tagged proteins at different concentrations. Top: extruded Par j 4; bottom: unrelated his-tagged protein. Also extruded Par j 4 collected between 0.4 and 2 ml is shown (Par j 4 EVs peak). Proteins are subjected to the treatment described in Materials and Methods (2.10 Immunodot blotting). The figure shows that the capability of protein detection by the chemolumiscent probe is not altered by the applied treatment.
